# Anetoderma in a patient with a history of primary syphilis

**DOI:** 10.1002/ccr3.3290

**Published:** 2020-09-07

**Authors:** Sepehr Hamidi, Sharona Yashar

**Affiliations:** ^1^ Department of Pathology University of California Los Angeles CA USA; ^2^ Department of Dermatology West Los Angeles ‐ Veterans Affairs Hospital (WLA‐VA) Los Angeles CA USA

**Keywords:** Anetoderma, syphilis

## Abstract

Anetoderma is a rare cutaneous disorder presenting with atrophic skin lesions. It can be associated with several autoimmune and infectious diseases. With the current resurgence of syphilis, clinicians must be aware of its association with anetoderma.

## Graphical abstract

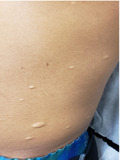

## 1 CASE REPORT

Anetoderma is a rare cutaneous disorder histologically characterized by localized loss or reduction of dermal elastic fibers. Syphilis, once the most common cause of anetoderma in the early 20th century, is making a worldwide comeback. In any patient with anetoderma, syphilis should be investigated as one of the differentials.

**FIGURE 1:**
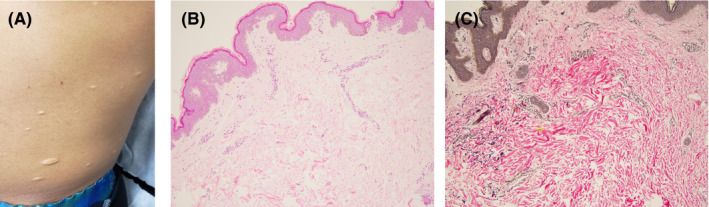
A, Multiple skin‐colored atrophic macules and papules. B, Hematoxylin and eosin stain, ×100. C, Elastic stain (Verhoeff‐Van Gieson, ×100) confirms significant loss of the elastic fibers in the affected dermis (center and right). Note the normal distribution of these fibers in the uninvolved dermis (left)

A 24‐year‐old man presented with multiple wrinkled, atrophic macules on his trunk, ranging from 0.5 to 1.5 cm (Figure 1A). His past history was only significant for primary syphilis 2 years ago which was treated with penicillin. There was no history of secondary syphilis or any other reported conditions.

A biopsy from one of his lesions was essentially unremarkable, showing only minimal superficial perivascular lymphocytic infiltrate on routine histological examination (Figure 1B). An elastic stain demonstrated a nearly complete loss of elastic fibers in superficial and mid‐dermis in the affected area (Figure 1C).

A diagnosis of anetoderma (also known as macular atrophy) was made. Anetoderma is characterized by a decrease of dermal elastic fibers; however, its precise pathophysiology remains unclear.^1^ Histological appearance of the biopsy can be essentially normal on routine H&E‐stained sections. Clinical correlation and performing special stains are necessary for diagnosis. Anetoderma may be primary or it can be secondary and arise at the same sites of prior cutaneous lesions. In the absence of any previous skin pathology including secondary syphilis, the anetoderma in this patient was considered primary. Both primary and secondary anetoderma can develop in patients with a history of syphilis within months or years after the infection.^2^ Due to its association with several autoimmune disorders (eg, lupus erythematosus), infections, and medications, a thorough clinical and laboratory workup is necessary once anetoderma is diagnosed. There is no effective treatment.

## CONFLICT OF INTEREST

None declared.

## AUTHOR CONTRIBUTIONS

SH: did literature review, drafted the manuscript, and is the corresponding author. SY: is the dermatologist who made the diagnosis, took the clinical history, and performed patient care.
